# Scaled traumatic brain injury results in unique metabolomic signatures between gray matter, white matter, and serum in a piglet model

**DOI:** 10.1371/journal.pone.0206481

**Published:** 2018-10-31

**Authors:** Emily W. Baker, W. Matthew Henderson, Holly A. Kinder, Jessica M. Hutcheson, Simon R. Platt, Franklin D. West

**Affiliations:** 1 Regenerative Bioscience Center, University of Georgia, Athens, GA, United States of America; 2 Department of Animal and Dairy Science, University of Georgia, Athens, GA, United States of America; 3 National Exposure Research Laboratory, Office of Research and Development, United States Environmental Protection Agency, Athens, GA, United States of America; 4 Department of Small Animal Medicine and Surgery, University of Georgia, Athens, GA, United States of America; University of Florida, UNITED STATES

## Abstract

Traumatic brain injury (TBI) is a leading cause of death and long-term disability in the United States. The heterogeneity of the disease coupled with the lack of comprehensive, standardized scales to adequately characterize multiple types of TBI remain to be major challenges facing effective therapeutic development. A systems level approach to TBI diagnosis through the use of metabolomics could lead to a better understanding of cellular changes post-TBI and potential therapeutic targets. In the current study, we utilize a GC-MS untargeted metabolomics approach to demonstrate altered metabolism in response to TBI in a translational pig model, which possesses many neuroanatomical and pathophysiologic similarities to humans. TBI was produced by controlled cortical impact (CCI) in Landrace piglets with impact velocity and depth of depression set to 2m/s;6mm, 4m/s;6mm, 4m/s;12mm, or 4m/s;15mm resulting in graded neural injury. Serum samples were collected pre-TBI, 24 hours post-TBI, and 7 days post-TBI. Partial least squares discriminant analysis (PLS-DA) revealed that each impact parameter uniquely influenced the metabolomic profile after TBI, and gray and white matter responds differently to TBI on the biochemical level with evidence of white matter displaying greater metabolic change. Furthermore, pathway analysis revealed unique metabolic signatures that were dependent on injury severity and brain tissue type. Metabolomic signatures were also detected in serum samples which potentially captures both time after injury and injury severity. These findings provide a platform for the development of a more accurate TBI classification scale based unique metabolomic signatures.

## Introduction

Traumatic brain injury (TBI) is a major public health issue with approximately 1.7 million people sustaining a TBI annually in the United States and one third of survivors experiencing long-term disability [1, 2]. Despite advancements in understanding the underlying mechanisms of TBI, there has been no significant progress in clinical treatment [3]. In clinical trials, it is common for TBI cases to be broadly classified as mild, moderate, or severe based on the Glasgow Coma Scale (GCS) which measures the level of consciousness after traumatic insult [4]. However, the GCS is a gross assessment that does not account for the pathoanatomic features responsible for the measured neurological deficits; therefore two TBI cases with divergent injury characteristics and outcomes could be identically classified [3]. To combat this, attempts have been made to develop scales based on other non-invasive modalities such as computed topography (CT) to identify pathoanatomic characteristics (e.g. midline shift, lesion size, the presence of hemorrhage, and cistern alterations) to better classify TBI [5–7]. However, it is possible for brain images to appear normal despite metabolic abnormality and the presence of clinical symptoms [8]. Thus, the heterogeneity of the disease coupled with the lack of standardized scales to properly categorize multiple types of TBI remains to be a major challenge facing the development of effective TBI treatments.

Development of more comprehensive diagnostic tools that measure deficits at the cellular level may enable better classification of patients based on TBI type and severity, potentially providing a predictive tool of outcome and ultimately aid in the development of therapeutic treatment plans. While the quantification of TBI biomarkers such as glutamate, glucose, and S100-B in serum, plasma, or cerebrospinal fluid (CSF) provide greater insight to the physiological alterations resulting from TBI, the clinical management and assessment of these metabolites remains unreliable especially in the case of mild TBI; furthermore, the focus on discrete molecules does not account for complex cellular interactions [9–13]. Emerging tools in the field of metabolomics provides an excellent platform to obtain more robust and comprehensive information about injury status or likelihood of therapeutic benefit based on a signature of markers rather than a single biomarker [9, 13, 14]. This systems level approach to TBI diagnosis could lead to a better understanding of the complex cascades of interacting metabolic changes after TBI and potential therapeutic targets.

Metabolomics is the study of the metabolites and metabolism within a biological system, which is a product of both the organism’s genome and the influence of its environment [14]. Analytical chemistry techniques such as gas chromatography coupled with mass spectrometry (GC-MS) are used to identify the constituents of complex biological mixtures with high sensitivity [14]. A major advantage to MS-based metabolomics is that an untargeted, “bottom-up” approach can be utilized in an attempt to quantify all biomolecules within a sample rather than quantifying a predetermined list of metabolites; therefore, it is possible to obtain the metabolomic signature for a particular patient or discover novel biomarkers for injury or disease [13, 15]. Microarray analysis in rodent TBI models have shown that the brain undergoes abundant changes in gene expression that are dependent on experimental conditions such as brain tissue type, time after injury, and injury severity, providing further evidence that the heterogenic nature of TBI results in a unique metabolomic signature for each TBI case [16–19]. Thus, metabolomics offers the possibility integrating “personalized medicine” into clinical TBI management by customizing a patient’s medical treatment based on their specific biomarker profile [14]. A major component of the pathophysiologic response to TBI is brain region dependent and more studies are urgently needed to compare the metabolomic response of gray and white matter after TBI. Gray and white matter compartments have distinctive cellular composition and cytoarchitecture leading to differing responses to neural injury with respect to structural vulnerability, susceptibility to excitotoxic and ischemic insults, and edema accumulation [20–25]. Preclinical animal models of TBI that possess a similar gray to white matter ratio as humans, such as the pig, will enable the assessment and understanding of large scale region-specific metabolic responses to neural injury that are likely to be more clinically relevant. Furthermore, the pig has greater metabolomic similarity to other common laboratory animals [26]. Together, this evidence suggests that a pig TBI model could lead to a better understanding of TBI-induced, region-specific metabolic alterations in the human brain compared to traditional rodent systems.

We have recently demonstrated that increasing controlled cortical impact (CCI) parameters, velocity and depth of depression, directly correlate with increased lesion size, neuronal loss, astrogliosis/astrocytosis, and white matter damage [27]. These graded cellular and tissue level changes ultimately led to increased motor function deficits with changes in stride velocity and gait parameters associated with stability. However, questions still remained pertaining to the metabolic changes that occur at the cellular level with increasing TBI injury. In the current study, we utilize an untargeted GC-MS metabolomics approach to demonstrate variable alterations in brain metabolism after scaled TBI in a pig model. Pathway analysis reveals that gray and white matter are differentially affected by TBI in a severity-dependent manner. Furthermore, metabolomic analysis performed on serum 24 hours and 7 days post-TBI demonstrates a distinct time course of metabolic alteration that is also dependent on TBI severity but independent of alterations observed directly in brain tissue. This proof-of-concept study illustrates that untargeted metabolomic methodology captures unique signatures that are contingent on sample type (i.e. gray matter, white matter, or biofluid), TBI severity, and time after injury in a translational pig TBI model. These findings will potentially lead to a greater understanding of the intricate, heterogenic cellular changes after TBI, resulting in a more comprehensive and accurate classification system for a personalized medicine approach to TBI treatment.

## Materials and methods

### Animals

All work in this study was prospectively approved (identification number A2012 10-005-Y3-A7) by the University of Georgia Institutional Animal Care and Use Committee (UGA IACUC), and all studies were performed in adherence to the UGA IACUC guidelines set forth. Sixteen (n = 4 per injury severity group) commercially bred American Landrace piglets of mixed sex and three weeks of age were obtained from the University of Georgia Swine Unit one week prior to surgery. Piglets were group housed and fed a pelleted starter diet ad libitum. The room temperature was maintained at 26°C with a 12 hour light/dark cycle. An overhead heat lamp provided supplemental heat to a portion of the enclosure. Animals were feed restricted overnight prior to surgery but were permitted free access to water at all times.

### Controlled cortical impact

Concussive TBI was generated through the use of a specialized CCI device that was designed by our lab in collaboration with the University of Georgia Instrument Shop (Athens, GA) and was modeled after the design of Manley et al [28]. The CCI device is designed to generate a focal, reproducible injury to the cerebral cortex with defined impact parameters including impact velocity, depth of cortical depression, and dwell time. At four weeks of age, piglets underwent surgery to induce concussive TBI. Anesthesia was induced via inhalation of 5% vaporized isoflurane in oxygen utilizing a nose cone. After induction, anesthesia was maintained at 2–3% vaporized isoflurane in oxygen. Heart rate, respiration rate, and rectal temperature was measured every five minutes throughout the duration of surgery and post-operative recovery. The skin over the cranium was prepared in a routine manner for sterile surgery using Betadine and 70% alcohol, and the surgical site was draped in a standard fashion. A sagittal left-sided skin incision approximately four centimeters in length was made over the top of the cranium, and the periosteal layers were resected to expose the cranial bone. A dental air drill (Brasseler USA) was used to drill a circular craniectomy approximately 20mm in diameter at the left posterior junction of the coronal and sagittal sutures. The underlying dura mater was left intact. Anesthetized piglets were then secured to the CCI frame, and the impactor tip was positioned over the site of the cranioectomy. Piglets were divided into four treatment groups depending on the impact parameters in which TBI was induced, including: 2 m/s impact velocity with 6mm depth of depression (2m/s; 6mm, n = 4), 4 m/s impact velocity with 6mm depth of depression (4m/s; 6mm, n = 4), 4 m/s impact velocity with 12mm depth of depression (4m/s; 12mm, n = 4), 4 m/s impact velocity with 15mm depth of depression (4m/s; 15mm, n = 4). The dwell time, defined as the amount of time the impactor tip makes contact with the cortex before retraction, was held constant at 400 ms for all treatment groups. After TBI induction, the cranioectomy site was flushed with sterile saline and the skin was re-apposed with sterile surgical staples. Piglets were then removed from anesthesia and continuously monitored until ambulatory. Banamine (1.1 mg/kg IM) and butorphanol (0.2 mg/kg IM) was administered postoperatively for analgesia, and Ceftiofur sodium (Naxcel; 4 mg/kg IM) was administered postoperatively as an antibiotic.

### Serum and tissue sample preparation

Blood samples were collected pre-TBI, 24 hours post-TBI, and 7 days post-TBI from the lateral saphenous vein and allowed to coagulate in serum separator tubes (BD Vacutainer). Blood samples were then centrifuged at 2,000 rpm for 20 minutes, and the serum supernatant was collected, snap frozen in liquid nitrogen, and stored at -80°C. 7 days post-TBI, piglets were deeply anesthetized through inhaled 5% vaporized isoflurane in oxygen and immediately euthanized via CO_2_ asphyxiation following American Veterinary Medical Association guidelines. The piglets were decapitated and the brain was removed. Brain samples were collected from the peri-lesional area of the ipsilateral cortex and subcortical white matter, snap frozen in liquid nitrogen, and stored at -80°C until use. Four healthy, uninjured piglets of the same age were sacrificed and brain tissues were collected as described above to serve as normal controls.

### Metabolite extraction

For metabolite extraction, serum and tissue samples were thawed on ice. For each tissue sample, gray and white matter was grossly identified based on tissue color, and a sterile scalpel blade was used to separate gray matter from white matter. Approximately 65mg of gray and white matter was extracted separately. For serum, a volume of ~70 μL was used for extraction. Metabolites were extracted from the serum and tissue samples in centrifuge tubes using methanol, deionized water, and chloroform (ratio of 1.4:1:1.8). A 3.2-mm stainless steel bead (Biospec Products, Inc.) was added to each centrifuge tube and samples were homogenized using a Tissue lyser (Qiagen). As described previously [29], 400 μL of methanol, 285 μL of deionized water, and 500 μL cholorform was added stepwise to the samples. After centrifugation, the polar metabolite phase was removed, aliquoted into GC vials, and lyophilized in a Savant Speed Vac Concentrator (Thermo Scientific).

### Data acquisition and analysis of metabolites

Tissue samples were analyzed on a LECO Pegasus 4D GC-ToF/MS (St. Joseph, MI) interfaced to an Agilent 7890B gas chromatograph. Chromatographic separation was achieved on a DB-5ms (30m, 0.25 μm thickness, and 0.25 mm ID, Agilent Technologies, CA). The injector, transfer line and ion source were held at 275°C, 280°C and 225°C respectively. Samples were injected in splitless mode (2 μL) and helium maintained at a constant flow of 0.8 mL/min. The initial oven temperature was held for 2 min at 60°C and ramped at 8°C/min to 300°C and held for 5 min. Mass spectra were acquired from 50–650 *m/z* at an acquisition rate of 20 spectra/second. Serum samples were analyzed similarly on a Waters Autospec mass spectrometer using the same column and temperature profiles. For serum analysis, the trap was set at 350 μA and the detector at 300 V.

All chromatograms were then exported as netcdf files using the instrument specific software and imported into MetAlign for data alignment using recommended parameters for time of flight mass spectrometers [30]. After spectral alignment and normalization to TIC, data was filtered in Excel as described previously [31]. Student’s t-test filtered chromatograms comparing the response of TBI groups to control were constructed in Excel by subtracting the average response of control samples from TBI samples at each retention time (R.T.) m/z pair. SIMCA-P+ (Umetrics, Sweden) was used for partial least squares-discriminant analysis (PLS-DA), and MetaboAnalyst 3.0 used for ANOVA and pathway analysis as described below [32]. For each statistical platform, metabolite abundances were Pareto scaled prior to multivariate analysis. In SIMCA-P+, PLS-DA models were cross-validated using software recommended parameters and only models with a cumulative Q2 > 0.6 in the first three components were used for class visualization. Metabolites were identified by comparing the acquired spectra to commercially available databases (e.g. NIST 2014) and considered a putative identification with a similarity score greater than 700.

### Metabolic pathway analysis

As described in previous studies [33], the Pathway Analysis module on Metaboanalyst (http://www.metaboanalyst.ca) was used to identify affected metabolic pathways based on metabolites identified by GC-MS to be up- or downregulated after TBI. A *Sus scrofa*-specific library is not available at this time, so we selected the *Homo sapiens* library and used the default “Hypergeometric Test” and “Relative-betweenness Centrality” algorithms for over representation analysis and pathway topology analysis, respectively [34, 35]. In order to report the most relevant metabolic pathways, the impact value threshold was set to 0.1.

## Results

### Increasing TBI severity results in correlative PLS-DA group separation with distinct responses between gray and white matter

**Fig 1:**
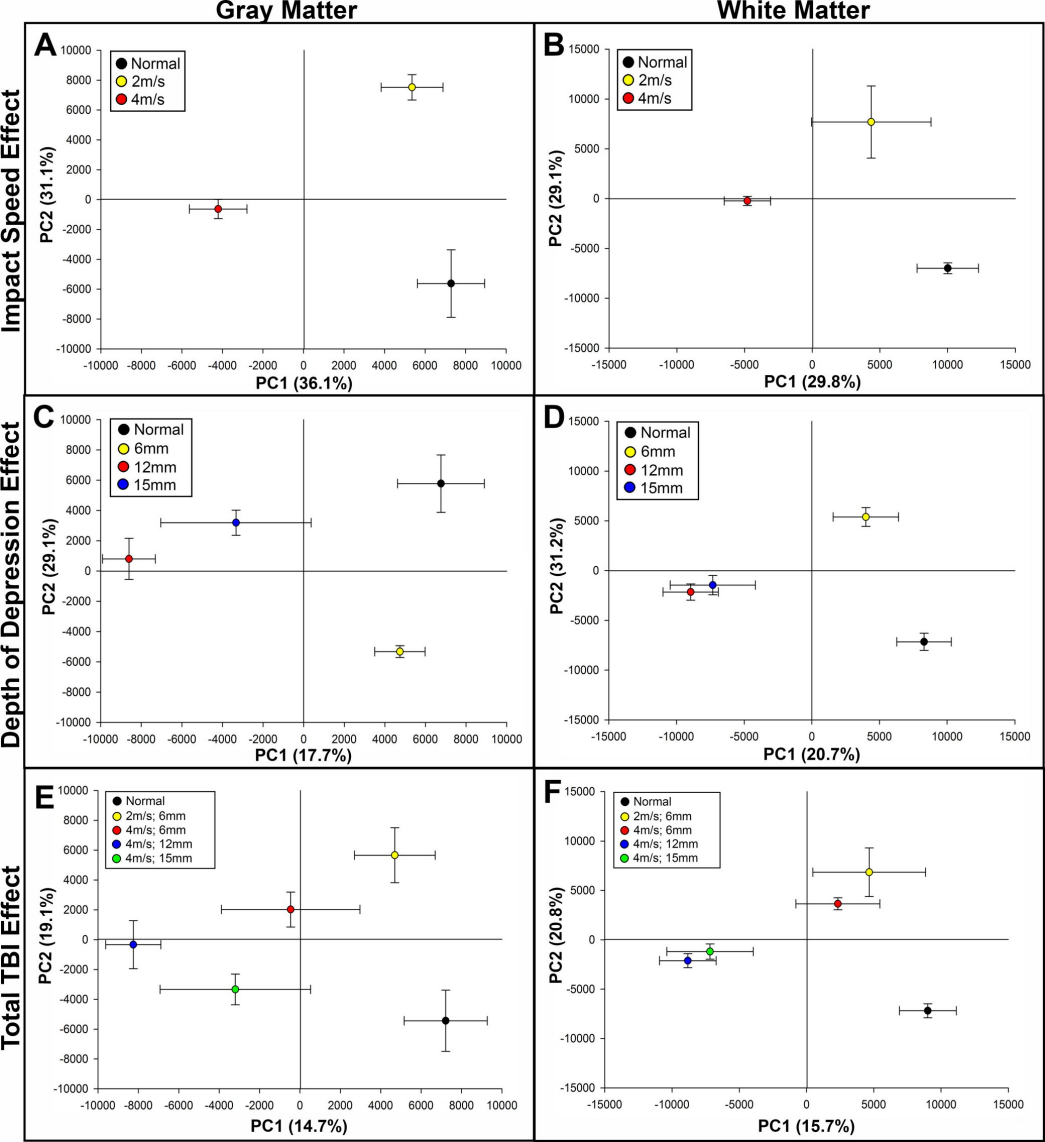
PLS-DA score plots illustrate distinct metabolomic separation based on TBI severity in gray and white matter. PLS-DA scores plots from analysis of the GC-MS chromatograms of gray (A, C, E) and white (B, D, F) brain matter 7 days post-TBI. Severity-dependent trajectories based on impact speed effect (A, B), depth of depression effect (C, D), and total TBI effect (E, F) are illustrated in each panel. PC1 = principal component 1, PC2 = principal component 2. R2Y values for PC1 and PC2 are illustrated as percentage in parentheses on each axes.

Partial least squares-discriminant analysis (PLS-DA) was utilized to compare the metabolomic profile of normal control brain tissue and brain tissue isolated from the peri-lesional brain region of pigs that sustained TBI with impact parameters of 2m/s; 6mm, 4m/s; 6mm, 4m/s; 12mm, and 4m/s; 15mm. We first assessed the influence of impact speed and depth of depression on group separation from normal control tissues and within TBI groups, and whether gray and white matter responded differently to changes in these impact parameters. While holding depth of depression constant at 6mm, pigs that sustained a TBI at 2m/s demonstrated a metabolomic profile more similar to normal control tissue compared to pigs that sustained a TBI at 4m/s in both gray and white matter along principal component 1 (PC1), which is illustrated by separation along the X axis, and this distance from control samples potentially represents the largest biological changes in the system (**Fig 1A and 1B**). Similarly, while holding impact speed constant at 4m/s, pigs that sustained a TBI with 6mm depth of depression were more similar to normal controls along PC1 compared to pigs that sustained a TBI at 12mm or 15mm in both gray and white matter; however, the 12mm and 15mm groups displayed less separation in white matter compared to the response in gray matter (**Fig 1C and 1D**). PLS-DA of total TBI effect demonstrated correlative group separation in gray and white matter with increasing TBI severity along PC1 (**Fig 1E and 1F**). However, while a stepwise increase in group separation between TBI groups along PC1 was documented in gray matter, group clustering was apparent in white matter TBI groups in which the 2m/s; 6mm and 4m/s; 6mm groups were clustered together, and the 4m/s; 12mm and 4m/s; 15mm groups were clustered together while maintaining distinct separation from the less severe groups (**Fig 1F**). Taken together, this data indicates that both impact velocity and depth of depression play a role in changing the metabolomic profile after TBI, and gray and white matter may respond differently to TBI on the metabolic level.

### White matter displays a greater number of metabolomic alterations than gray matter after TBI

**Fig 2:**
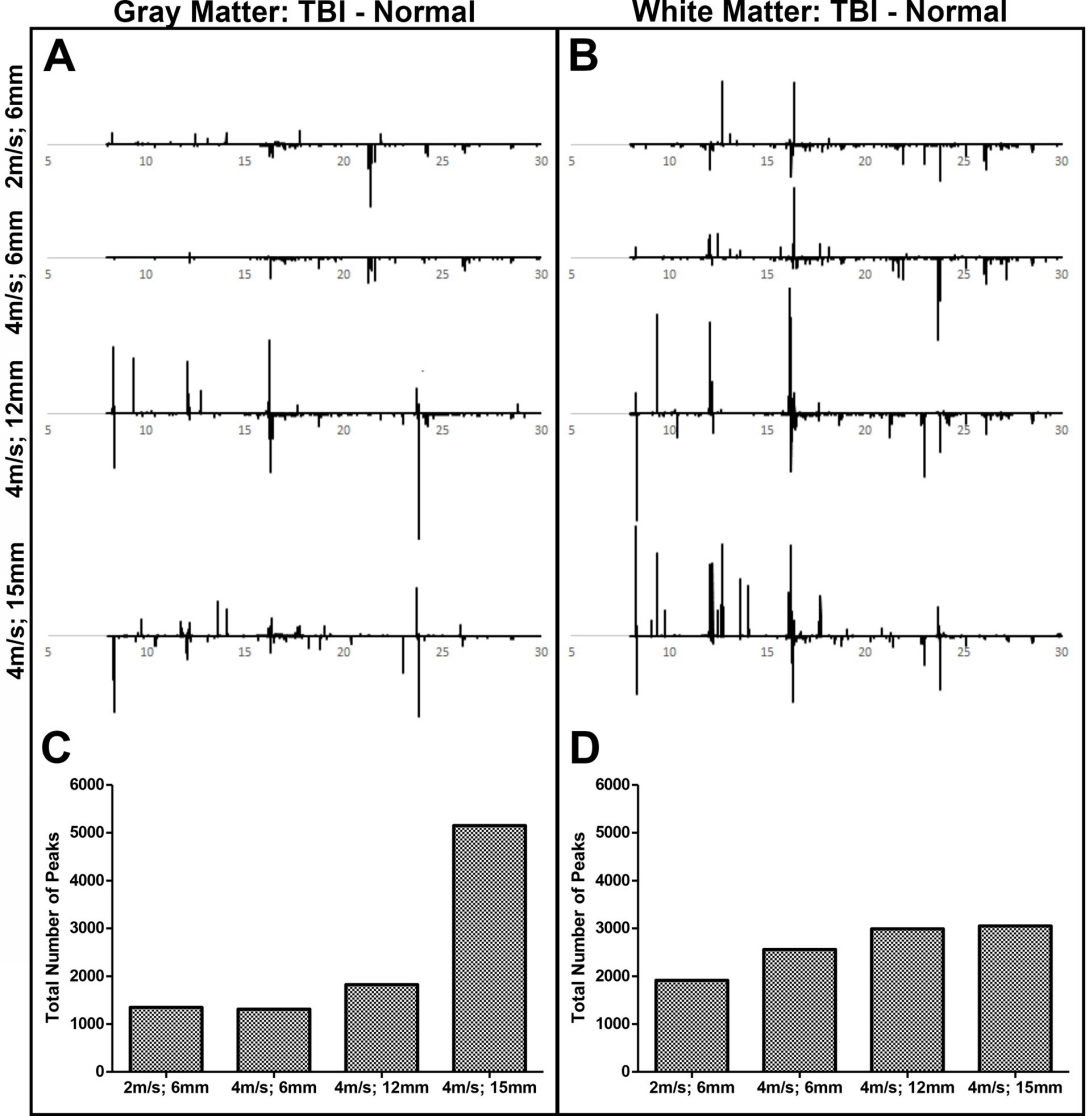
White matter displays greater metabolomic alterations than gray matter after TBI. *T*-test filtered difference chromatograms (p ≤ 0.05) illustrating the metabolomic response of gray (A) and white (B) brain matter 7 days post-TBI compared to normal control brain tissue. Y-axis values were held constant to aid in the visualization of relative changes in metabolite concentrations across groups. Peaks above baseline correspond to metabolites that were significantly increased following TBI compared to normal controls, and peaks below baseline correspond to metabolites that were significantly decreased following TBI compared to normal controls (A, B). After peak deconvolution and alignment of each individual animal, the absolute count of spectral features (i.e. R.T./m/z pairs) shown to be significantly increased or decreased compared to normal controls based on t-test analysis was calculated for each TBI group in gray (C) and white (D) matter.

Univariate analysis was performed to quantify significant changes in metabolite abundance in TBI tissue compared to normal control tissue in both gray and white matter. In this visualization (**Fig 2A and 2B**), peaks in the negative direction (less than 0) represent metabolites that are downregulated (i.e. higher in control samples) after TBI while peaks in the positive direction (more than 0) represent metabolites that are upregulated after TBI. It should be noted that MetAlign allows for spectral deconvolution and multiple ‘peaks’ can represent a single metabolite thus we use ‘peak’ and spectral features (i.e. R.T/m/z pairs) interchangeably. Overall, there was a positive correlation between TBI severity and the total number of spectral features (**Fig 2C and 2D**) as well as the total peak amplitude (data not shown) in both gray and white matter. Furthermore, the total number of peaks as represented by R.T./*m/z* pairs was higher in white matter compared to gray matter in the 2m/s; 6mm, 4m/s; 6mm, and 4m/s; 12mm groups (1915 vs. 1348, 2558 vs. 1312, and 2992 vs. 1825, respectively), while the total number of peaks was higher in gray matter compared to white matter in the 4m/s; 15mm group (5152 vs. 3051, respectively, **Fig 2C and 2D**). Peak identification demonstrated that multiple classes of metabolites were either up- or downregulated by TBI including biological acids, alcohols, amino acids, fatty acids, and sugars (**S1 Table**). This data further supports that increasing TBI severity results in a greater change in brain metabolism, and gray and white matter respond differently to TBI with white matter demonstrating more metabolic alterations compared to gray matter in all TBI groups with the exception of the most severe group.

### Pathway analysis reveals unique metabolomic signatures dependent on TBI severity and brain tissue type

Metaboanalyst was used to identify impaired metabolic pathways after TBI in gray and white matter. As expected, there were generally more affected metabolic pathways with increasing TBI severity (**Table 1**). Metabolic pathways, shown to be significantly altered based on a pathway impact threshold of 0.1 calculated by Metaboanalyst, demonstrated a distinct pattern of susceptibility across TBI severity and brain tissue type; some pathways (i.e. glycolysis, methane metabolism) were preferentially affected in gray matter while other pathways (i.e. cysteine and methionine metabolism, lysine degradation) were preferentially affected in white matter. Additionally, some pathways were only affected in more severe TBI groups (i.e. butanoate metabolism) while some pathways were affected across all TBI severities or brain tissue types (i.e. arginine and proline metabolism, galactose metabolism, etc). This data indicates that each TBI group demonstrates unique changes in metabolomic signature that is dependent on brain tissue type.

**Table 1:** Altered metabolic pathways after TBI in gray and white brain matter.

Pathway	GM 2m/s; 6mm	WM 2m/s; 6mm	GM 4m/s; 6mm	WM 4m/s; 6mm	GM 4m/s; 12mm	WM 4m/s; 12mm	GM 4m/s; 15mm	WM 4m/s; 15mm
**Citrate cycle (TCA cycle)**					**+**			
**Glycolysis or Gluconeogenesis**					**+**		**+**	
**Methane metabolism**					**+**		**+**	
**Glyoxylate and dicarboxylate metabolism**					**+**		**+**	
**Pyruvate metabolism**	**+**		**+**		**+**		**+**	
**Cysteine and methionine metabolism**				**+**		**+**		**+**
**Lysine degradation**		**+**		**+**		**+**		**+**
**Pantothenate and CoA biosynthesis**		**+**		**+**		**+**		
**Phenylalanine metabolism**		**+**		**+**		**+**		
**Starch and sucrose metabolism**				**+**		**+**		**+**
**Butanoate metabolism**					**+**	**+**	**+**	**+**
**Glycerolipid metabolism**		**+**		**+**	**+**	**+**	**+**	**+**
**Arachidonic acid metabolism**	**+**		**+**		**+**	**+**	**+**	**+**
**AminoacyltRNA biosynthesis**				**+**				**+**
**Alanine, aspartate and glutamate metabolism**	**+**	**+**	**+**	**+**	**+**			
**Arginine and proline metabolism**	**+**	**+**	**+**	**+**	**+**	**+**	**+**	**+**
**Galactose metabolism**	**+**	**+**	**+**	**+**	**+**	**+**	**+**	**+**
**Glycine, serine and threonine metabolism**	**+**	**+**	**+**	**+**	**+**	**+**	**+**	**+**
**Inositol phosphate metabolism**	**+**	**+**	**+**	**+**	**+**	**+**	**+**	**+**

*Note*: **+** = significantly altered after TBI based on impact-value threshold set to 0.1

### PLS-DA score plots display distinct group separation in serum samples based on TBI severity and time after injury

**Fig 3:**
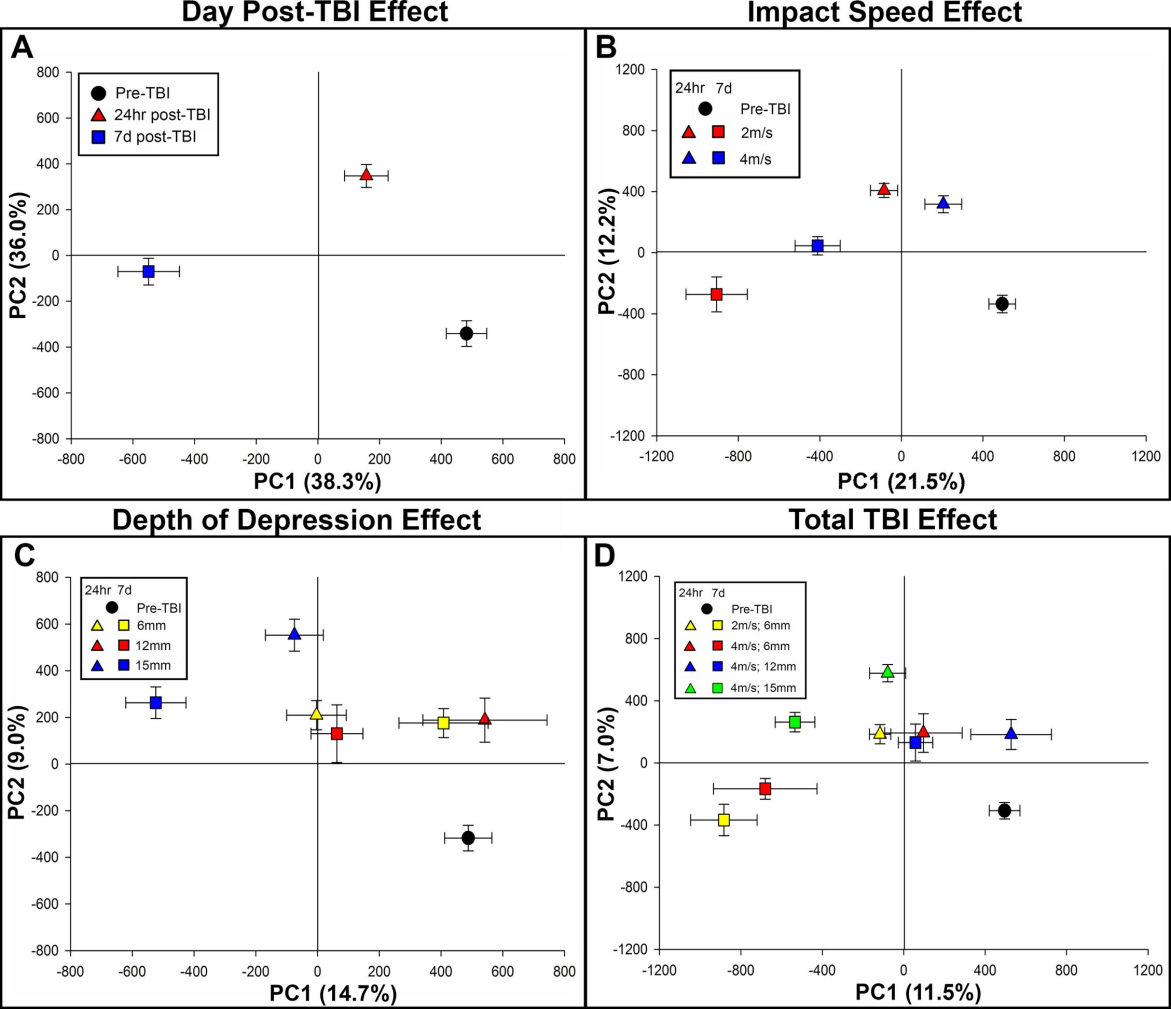
PLS-DA score plots displays distinct metabolomic separation in serum based on TBI severity and day post-TBI. PLS-DA scores plots from analysis of the GC-MS chromatograms of serum collected 24 hours and 7 days post-TBI. Trajectories based on day post-TBI (A), impact speed effect (B), depth of depression effect (C), and total TBI effect (D) are illustrated in each panel. PC1 = principal component 1, PC2 = principal component 2. R2Y values for PC1 and PC2 are illustrated as percentage in parentheses on each axes.

Next, we assessed the changes in the metabolomic profile of serum collected from piglets 24 hours and 7 days post-TBI in order to 1) determine the extent the metabolomic profile changes over time after TBI and 2) whether TBI-induced metabolic alterations differed between serum and brain tissue. When assessing the effect of time after injury regardless of injury severity, PLS-DA score plots revealed distinct group separation between the pre-TBI, 24 hours post-TBI, and 7 days post-TBI. Furthermore, the 24 hour post-TBI metabolomic profile was closer to pre-TBI along PC1 compared to the 7 days post-TBI metabolomic profile (**Fig 3A**). When assessing how metabolism is influenced by impact speed over time while holding depth of depression constant at 6mm, we found that serum collected 24 hours post-TBI was more similar to pre-TBI along PC1 for both 2m/s and 4m/s groups than either group 7 days post-TBI, which indicates that time post-TBI has a greater influence on the metabolomic profile of serum than impact speed (**Fig 3B**). When assessing the influence of depth of depression over time while holding impact speed constant at 4m/s, we found a stepwise increase in group separation from pre-TBI along PC1 7 days post-TBI as severity was increased (**Fig 3C**). Furthermore, distinct group separation was present when assessing total TBI effect across all TBI severities and time points. Interestingly, there was greater group separation across PC1 in serum from pigs in the 2m/s; 6mm and 4m/s; 6mm groups 7 days post-TBI compared to the serum collected from pigs at the same time point that sustained a more severe TBI, albeit less group separation was present along PC2 (Y axis) 7 days post-TBI in the 2m/s; 6mm and 4m/s; 6mm groups (**Fig 3D**). Together, this data indicates that time point, impact speed, and depth of depression all contribute to the altered metabolomic signature of serum collected post-TBI.

### Serum demonstrates dynamic changes in metabolomic profile in all TBI severities and over time

**Fig 4:**
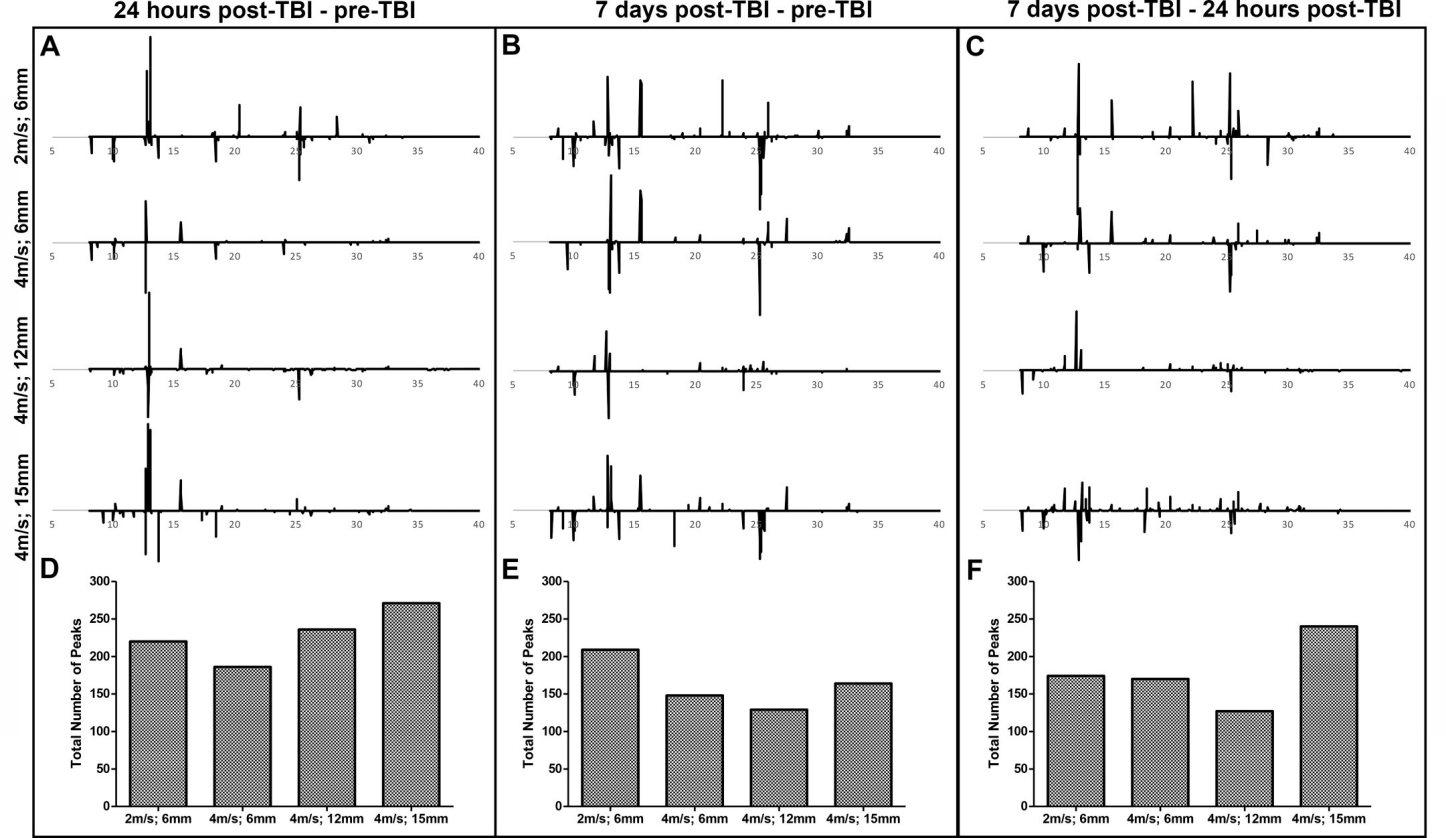
Serum demonstrates dynamic changes in metabolomic profile in all TBI severities and over time. *T*-test filtered difference chromatograms (p ≤ 0.05) illustrating the metabolomic differences 24 hours post-TBI compared to pre-TBI (A), 7 days post-TBI compared to pre-TBI (B), and 7 days post-TBI compared to 24 hours post-TBI (C). Y-axis values were held constant to aid in the visualization of relative changes in metabolite concentrations across groups. For A and B, peaks above baseline correspond to metabolites that were significantly increased following TBI compared to pre-TBI, and peaks below baseline correspond to metabolites that were significantly decreased following TBI compared to pre-TBI. For C, peaks above baseline correspond to metabolites that were significantly increased 7 days post-TBI compared to 24 hours post-TBI, and peaks below baseline correspond to metabolites that were significantly decreased 7 days post-TBI compared to 24 hours post-TBI (A-C). After peak deconvolution and alignment of each individual animal, the total number of peaks, or spectral features, above/below baseline were calculated for each TBI group within the 24 hours post-TBI vs. pre-TBI (D), 7 days post-TBI vs. pre-TBI (E), and 7 days post-TBI vs. 24 hours post-TBI (F) comparisons.

Univariate analysis was performed on serum collected pre-TBI, 24 hours post-TBI, and 7 days post-TBI across TBI groups to quantify changes in the metabolomic profile between TBI groups and over time. In **Fig 4A** and **4B**, peaks in the negative direction (less than 0) represent metabolites that are downregulated 24 hours and 7 days post-TBI, respectively, compared to pre-TBI while peaks in the positive direction (more than 0) represent metabolites that are upregulated after TBI compared to pre-TBI. In **Fig 4C**, peaks in the negative direction (less than 0) represent metabolites that are downregulated 7 days post-TBI compared to 24 hours post-TBI while peaks in the positive direction (more than 0) represent metabolites that are upregulated 7 days post-TBI compared to 24 hours post-TBI. At 24 hours post-TBI, there was a correlative increase in total number of peaks and the total peak amplitude (data not shown) as TBI severity was increased (**Fig 4D**). However, when the absolute number of significantly changed peaks was compared to 7 days post-TBI, there was a correlative decrease in the total number of significant peaks as TBI severity was increased (**Fig 4E**). The differences in correlation to TBI severity between the two time points are indicative of distinct metabolic alterations between 24 hours and 7 days post-TBI. Indeed, univariate analysis comparing 7 days post-TBI and 24 hours post-TBI revealed many peaks above/below baseline indicative of altered metabolite abundance between the two time points in the 2m/s; 6mm, 4m/s; 6mm, 4m/s; 12mm, and 4m/s; 15mm groups (174, 170, 127, and 240, respectively, **Fig 4F**). Furthermore, the total number of peaks was declined between 24 hours and 7 days post-TBI when both are compared to pre-TBI in the 2m/s; 6mm, 4m/s; 6mm, 4m/s; 12mm, and 4m/s; 15mm groups (220 vs. 209, 186 vs. 148, 236 vs. 129, and 271 vs. 164, respectively, **Fig 4D and 4E**). Serum metabolite classes similar to brain tissue where determined by peak identification to be upregulated or downregulated by TBI including acids, alcohols, amino acids, fatty acids, and sugars (**S2 Table**). This data indicates that serum undergoes dynamic changes in metabolism over time evident by altered peak abundance between pre-TBI, 24 hours post-TBI, and 7 days post-TBI.

### Pathway analysis of serum supports observed severity and time-dependent metabolomic alterations

Metabolic pathways that were affected by TBI were identified using Metaboanalyst in serum collected 24 hours and 7 days post-TBI. As shown in **Table 2**, a distinct pattern of pathway alterations was present based on TBI severity and time post-TBI. Glycerophospholipid metabolism was affected 24 hours post-TBI across all TBI severities while cysteine and methionine metabolism, phenylalanine metabolism, and aminoacyltRNA biosynthesis was affected exclusively 7 days post-TBI. Furthermore, a subset of pathways (i.e. citrate cycle, inositol phosphate metabolism, and butanoate metabolism) were affected 7 days post-TBI in only the more severe TBI groups. In contrast, another subset of pathways (i.e. alanine, aspartate, and glutamate metabolism and arginine and proline metabolism) were affected in nearly all TBI groups and time points. Taken together, the data indicates that serum collected post-TBI, like brain tissue, possesses a unique metabolomic signature dependent on both TBI severity and time post-TBI.

**Table 2:** Altered metabolic pathways after TBI in serum 24 hours and 7 days post-TBI.

Pathway	2m/s; 6mm 24 hours	2m/s; 6mm 7 days	4m/s; 6mm 24 hours	4m/s; 6mm 7 days	4m/s; 12mm 24 hours	4m/s; 12mm 7 days	4m/s; 15mm 24 hours	4m/s; 15mm 7 days
**Glycerophospholipid metabolism**	**+**		**+**		**+**		**+**	
**Cysteine and methionine metabolism**		**+**				**+**		**+**
**Phenylalanine metabolism**		**+**		**+**				**+**
**AminoacyltRNA biosynthesis**		**+**		**+**				
**Pyruvate metabolism**	**+**	**+**	**+**			**+**		**+**
**Glycine, serine, and threonine metabolism**	**+**	**+**		**+**			**+**	**+**
**Pyrimidine metabolism**			**+**					
**Galactose metabolism**	**+**						**+**	
**Lysine degradation**		**+**		**+**	**+**		**+**	
**Citrate cycle (TCA cycle)**						**+**		**+**
**Inositol phosphate metabolism**								**+**
**Butanoate metabolism**								**+**
**Alanine, aspartate, and glutamate metabolism**	**+**	**+**	**+**	**+**	**+**	**+**		**+**
**Arginine and proline metabolism**	**+**	**+**	**+**	**+**	**+**	**+**	**+**	

*Note*: **+** = significantly altered after TBI based on impact-value threshold set to 0.1

## Discussion

Despite advancements in understanding the cellular mechanisms of TBI, there are no reliable classification scales to accurately categorize this heterogenic disease to predict patient outcomes or develop potential therapeutics. Here we utilize an untargeted GC-MS metabolomics approach to show that distinct metabolic alterations occur after TBI which are dependent on injury severity, sample type, and time after injury. PLS-DA score plots of brain tissue isolated from the peri-lesional area demonstrated distinct group separation correlative to TBI severity, and these trajectories were different between gray and white matter. Interestingly, the total number of peaks (deconvoluted spectral features) quantified from *t*-test filtered difference chromatograms revealed that more metabolic alterations occur in white matter than gray matter when compared to their respective controls, which provides further justification to study TBI pathophysiology in preclinical animal models with comparable white matter to humans. Furthermore, metabolic changes measured in serum after TBI revealed dynamic alterations in metabolic pathways with a distinct time course. These results provide evidence that a TBI classification scale based on the global metabolome could be developed to predict TBI severity and brain structures affected in order to more accurately predict patient outcomes and identify therapeutic targets in a personalized manner.

### Unique metabolic alterations between gray and white matter

Untargeted metabolomic assessment of TBI-affected brain tissue demonstrated unique alterations in multiple metabolic pathways based on injury severity and tissue type. Previous metabolomics studies utilizing magnetic resonance approaches have quantified specific metabolites in tissue, cerebrospinal fluid (CSF), and plasma after TBI in order to identify predictors for cellular mechanisms or other clinical measures [36–40]. However, since these studies only target a predetermined list of metabolites, they may not identify novel markers or account for important interactions between molecules at the cellular level [12]. To overcome this limitation, previous studies have performed untargeted metabolomics on serum samples in order to classify TBI patients based on injury severity and the presence of cognitive impairment based on altered metabolomic signature [33, 41]. However, to our knowledge no studies have analyzed the effect of TBI on the global metabolome directly on brain tissue nor assessed differences in metabolic alteration between gray and white matter as we have done in this study. We utilized the Pathway Analysis module on Metaboanalyst, which uses the KEGG metabolic database, to identify metabolic pathways altered by TBI [42]. In order to identify the most relevant alterations, the impact value threshold was set to 0.1, thereby identifying pathways in which ≥ 10% of relevant metabolite nodes are significantly altered by TBI. Pathway analysis revealed that gray and white matter responded differently to TBI across all injury severities, a result likely due to the divergent cellular composition of the two compartments. While neuronal cell bodies, dendrites, and synapses are mostly restricted to gray matter compartments, white matter consists of myelinating oligodendrocytes and axon bundles that foster communication between neurons [43–45]. We found that the metabolic pathways related to cysteine and methionine metabolism, lysine degradation, pantothenate and CoA biosynthesis, and phenylalanine metabolism are specifically impaired in white matter after TBI, while showing no changes in gray matter. Furthermore, the citrate cycle, glycolysis/gluconeogenesis, methane metabolism, and glyoxylate and dicarboxylate metabolism were only affected in more severely impaired gray matter while pyruvate metabolism was affected in gray matter regardless of TBI severity. This suggests that gray and white matter undergo different metabolic processes in response to TBI, indicating the potential need to develop treatment interventions specific to the brain region being affected.

### Metabolic alterations in white matter post-TBI

The pathways shown to be altered in white matter directly involve the amino acids serine, methionine, cysteine, alanine, lysine, phenylalanine, and tyrosine along with the amino acid derivative cystathionine. Previous studies have measured the abundance of these amino acids in brain tissue, serum, plasma, and CSF after TBI with variable findings dependent on sample type and time after injury [33, 36, 37, 39, 40, 46]. However, to our knowledge, no studies have elucidated the differences in the response of these amino acids between gray and white matter after TBI. Similar to our study, Irie et al. showed a significant reduction in lysine, methionine, phenylalanine, and tyrosine 24 hours post-stroke in both the striatum and cortex utilizing LC-MS in a rat model while an ex vivo NMR study showed elevated levels of lysine, phenylalanine, and tyrosine in CSF collected 7 days post-TBI [39, 47]. These studies support our finding that phenylalanine and tyrosine levels are decreased in tissue but elevated in biofluid collected 7 days post-TBI. This suggests that in the event of TBI, intracellular amino acids are released into the extracellular space and diffuse into circulating biofluids. Furthermore, the observed amino acid response to TBI was different between gray and white matter samples suggestive of a divergent time course or susceptibility to amino acid alterations between the two tissues. Indeed, a study by Shimada et al. found that amino acids diffuse slowly from gray matter compartments into adjacent white matter and later into the CSF [48]. While we documented a reduction in alanine across all TBI severities and a reduction in serine in more severe TBI groups, previous studies have shown no difference in alanine and serine levels in tissue or CSF after TBI compared to controls at <24 hours and 7 days post-TBI [36, 37, 40]. The difference in the measured alanine and serine response may be due to injury severity or the sensitivity of the analytical method as these studies were collected using less sensitive magnetic resonance approaches rather than GC-MS [14].

### Metabolic alterations in gray matter post-TBI

The pathways specifically altered in gray matter are implicated in cellular respiration and involve the metabolites pyruvic acid, succinic acid, acetylaldehyde, ethanol, lactic acid, glucose, acetic acid, formic acid, glycine, serine, and oxalic acid, which were almost exclusively downregulated after TBI. Cellular respiration occurs in mitochondria which are highly mobile in both anterograde and retrograde directions in neurons and tend to accumulate in segments with high metabolic demand [49–51]. Mitochondria are abundant in the neuronal cell bodies, dendrites, and axon terminals where they produce ATP to maintain the ionic gradient, and thus neuronal excitability, while also facilitating presynaptic vesicle packaging and postsynaptic protein turnover which exclusively occurs in gray matter compartments [45, 50–52]. Therefore, our finding of altered cellular respiration function in gray matter is indicative of decreased ATP synthesis in neurons and suggests impaired synaptic activity. Reductions in glucose consumption and cerebral energy state (measured by ATP or lactate/pyruvate ratio) are hallmarks of TBI and are directly correlated to patient outcome [53]. The lactate/pyruvate ratio (LPR) has been used extensively in microdialysis monitoring of TBI patients and reflects energy crisis with increases in LPR indicating loss of oxygen in tissue and the switch from aerobic to anaerobic respiration [54, 55]. Although we did not directly quantify LPR in the present study, we documented deficits in metabolites implicated in both aerobic and anaerobic respiration (including lactic acid) in the peri-lesional gray matter by 7 days post-TBI. However, serum lactic acid levels were elevated 24 hours post-TBI in a subset of TBI groups. This finding supports previous clinical studies which report high lactate levels due to cerebral and corporal hyperglycolysis in the initial post-injury stage that become slightly depressed in the days following TBI [56–58]. Taken together, this suggests that TBI with gray matter involvement may be more likely to respond to therapies targeted at restoring cerebral energy homeostasis.

### Changes in metabolic profile correlate with injury severity

Pathway analysis also illustrated graded metabolic response correlative to injury severity in a subset of metabolic pathways. Butanoate metabolism was impaired specifically in the 4m/s; 12mm and 4m/s; 15mm groups in both gray and white matter, partly due to decreased levels of butyric acid. Butyric acid has been identified as a potential therapeutic for neurological disease through its ability to serve as a histone deacetylase inhibitor, energy substrate, and G protein-coupled receptor activator; therefore, deficits in butyric acid abundance could be detrimental in the context of brain injury [59]. Interestingly, in the 2m/s; 6mm and 4m/s; 6mm groups, glycerolipid metabolism was only altered in white matter while arachidonic acid metabolism was only affected in gray matter; however, both metabolic pathways were affected in both tissue types in the more severe TBI groups. This suggests that white and gray matter are more susceptible to alterations in glycerolipid and arachidonic acid metabolism, respectively. Alterations in glycerolipid metabolism were driven by the altered abundance of glycerol, which is a component of the myelin sheaths found in white matter compartments [60]. These findings suggest that glycerol levels, and thus glycerolipid metabolism, are altered at a lower threshold in white matter compared to gray matter due to the disruption of myelin sheaths. Furthermore, arachidonic acid has been shown to play a role in the production of reactive oxygen species (ROS) after TBI [61–63]. Since mitochondria are more abundant in gray matter and are a major source of ROS, this finding indicates that arachidonic acid metabolism is affected at a lower injury severity in gray matter compared to white matter [45, 51, 62]. Together, this indicates that metabolomic analysis in our pig TBI model is able to delineate the individual metabolic vulnerabilities of gray and white matter through changes in compartment-specific cellular processes.

### Serum metabolic signatures capture unique changes in time and injury severity

Serum samples demonstrated dynamic alterations in metabolism between 24 hours and 7 days post-TBI. Glycerophospholipid metabolism was shown to be significantly altered in serum collected 24 hours post-TBI in all TBI groups while there was no change compared to controls by 7 days post-TBI in serum or tissue samples. Glycerophospholipids are a major component of neural cell membranes and have been shown to be altered in a number of neurological disorders including TBI due to the disruption of these membranes [64, 65]. In the acute stage of TBI, excessive release of glutamate initiates an excitotoxic cascade, and the resulting buildup of intracellular Ca^2+^ activates phospholipases which degrade neuronal cellular membranes [66]. Therefore, our data provides evidence of excitotoxicity-induced neuronal membrane degradation by 24 hours post-TBI that becomes diminished by 7 days post-TBI when excitoxicity has largely subsided [67]. Conversely, a number of metabolic pathways were disturbed in serum collected 7 days post-TBI that were not found at 24 hours, which coincided with documented metabolic deficits in tissue collected at the same time point. However, the injury severity at which these pathways were affected varied between tissue and serum. For example, aminoacyl tRNA biosynthesis was found to be altered in serum collected from the 2m/s;6mm group but no change was detected in tissue from this TBI group. Aminoacyl tRNAs are produced by aminoacyl tRNA synthases, and the expression of the pro-inflammatory mediator macromolecular tRNA synthase complex-associated protein (MSC^p43^) has been shown to be biomarker in brain injury to delineate between traumatic and ischemic brain injuries [68, 69]. Therefore, this finding indicates that alterations in this pathway detected in serum could be utilized as a marker for brain injury in the context of mild TBI. Furthermore, two metabolic pathways were shown to be significantly affected in serum collected both 24 hours and 7 days post-TBI including arginine and proline metabolism. The documented deficits in arginine and proline metabolism were due in part to alterations in proline levels as well as glutamine and ornithine which are metabolites of arginine [70]. Arginine is a precursor to the free radical nitric oxide (NO), and it has been shown that arginine levels in plasma and CSF are inversely correlated to NO levels; therefore, plasma levels of arginine have been proposed as a biomarker for ischemic brain damage [71, 72]. Similar to our results, Louin et al. and others documented no change in arginine levels but decreased proline levels after TBI in a rat model which was correlative to increased NO levels, indicating that proline may be a better biomarker than arginine in the context of TBI [70, 73]. However, proline can be a precursor for arginine and may be utilized to maintain arginine levels in brain tissue; therefore, it may be more beneficial to assess the entire metabolic pathway rather than the level of a single component [70]. Our results indicate that this metabolic pathway as a whole could be used as a biomarker for TBI of all severities from 24 hours post-TBI to 7 days post-TBI. Together, pathway analysis of serum illustrates the presence of a unique metabolic signature that captures both the time after injury and injury severity.

## Conclusions

In conclusion, we utilized an untargeted metabolomics approach to show that distinct alterations in metabolism occur after TBI that are dependent on injury severity, sample type, and time after injury in a translational pig model. Pathways such as arginine/proline, galactose, glycine/serine/threonine, and inositol phosphate metabolism were commonly affected in tissue while alanine/aspartate/glutamate and arginine/proline metabolism were affected across all injury severities in serum. These common pathways may be a major target for therapeutic intervention for TBI. Furthermore, we show for the first time that divergent changes in metabolism occur between gray and white matter after TBI with evidence of white matter displaying a greater number of metabolic changes. These findings provide a platform for the development of a more accurate and comprehensive TBI classification scale based unique metabolic signatures, creating a new avenue for personalized medicine in the management and treatment of TBI.

## Supporting information

S1 Table Metabolites identified as significantly changed after TBI in gray and white brain matter. *Note*: ↓ = metabolite decreased after TBI; ↑ = metabolite increased after TBI.
(DOCX)

S2 Table Metabolites identified as significantly changed in serum 24 hours and 7 days post-TBI. *Note*: ↓ = metabolite decreased after TBI; ↑ = metabolite increased after TBI.
(DOCX)
