# Supplementary material for: Scaled traumatic brain injury results in unique metabolomic signatures between gray matter, white matter, and serum in a piglet model
Source: PLoS One. 2018 Oct 31;13(10):e0206481. doi: 10.1371/journal.pone.0206481 (PMC6209298; doi:10.1371/journal.pone.0206481)
Supplement: S1 Table — Note: ↓ = metabolite decreased after TBI; ↑ = metabolite increased after TBI. (DOCX) [file pone.0206481.s001.docx]

| **Metabolite** | **GM 2m/s; 6mm** | **WM 2m/s; 6mm** | **GM 4m/s; 6mm** | **WM 4m/s; 6mm** | **GM 4m/s; 12mm** | **WM 4m/s; 12mm** | **GM 4m/s; 15mm** | **WM 4m/s; 15mm** |
| --- | --- | --- | --- | --- | --- | --- | --- | --- |
| **Acids** | | | | | | | | |
| **Acetic acid** | **↓** | **↓** | **↓** | **↓** | **↓** | **↓** | **↓** | **↓** |
| **Acetylaspartylglutamic acid** |  |  |  |  |  |  |  | **↓** |
| **Aminoadipic acid** | **↓** |  | **↓** |  | **↓** |  | **↓** |  |
| **Aminobutyric acid** |  | **↓** | **↓** | **↓** | **↓** | **↓** |  | **↓** |
| **Arachidonic acid** | **↓** |  | **↓** |  | **↓** | **↓** | **↓** | **↓** |
| **Butyric acid** | **↓** |  | **↓** | **↓** | **↓** | **↓** | **↑** | **↓** |
| **Deoxyerythronic acid** |  |  |  |  |  | **↓** |  | **↓** |
| **Gluconic acid** | **↓** |  | **↓** |  | **↓** |  | **↓** |  |
| **Glutaric acid** | **↑** |  | **↑** | **↓** | **↓** |  |  |  |
| **Hydroxydodecanedioic acid** |  |  | **↓** |  | **↓** |  | **↓** |  |
| **Hydroxypyruvic acid** | **↓** | **↓** | **↓** | **↓** | **↓** | **↓** | **↓** | **↓** |
| **Lactic acid** | **↓** |  |  |  | **↓** |  | **↓** |  |
| **Maleic acid** |  | **↓** |  | **↓** |  |  |  |  |
| **Malonic acid** |  | **↓** |  |  |  | **↓** |  |  |
| **Oxalic acid** |  |  |  |  | **↑** |  |  |  |
| **Phosphoric acid** |  | **↓** |  | **↓** | **↑** | **↓** | **↓** | **↓** |
| **Picolinic acid** |  |  |  |  |  | **↓** |  | **↓** |
| **Pipecolic acid** |  |  |  |  | **↓** |  | **↓** |  |
| **Pyrrole-2-carboxylic acid** |  | **↑** |  | **↑** | **↓** | **↑** | **↓** | **↑** |
| **Succinic acid** |  |  | **↓** |  | **↓** |  |  |  |
| **Terephthalic acid** |  |  |  |  | **↓** |  | **↓** |  |
| **Threonic acid** |  |  |  | **↓** | **↓** | **↓** | **↓** | **↓** |
| **Tricarballylic** **acid** |  |  |  |  |  | **↓** |  | **↓** |
| **Valeric acid** |  |  |  |  |  |  | **↓** |  |
| **Alcohols** | | | | | | | | |
| **Arabitol** |  |  |  | **↓** | **↓** | **↓** | **↓** | **↓** |
| **Butanol** | **↓** |  |  |  |  |  |  |  |
| **Erythritol** |  |  | **↓** |  | **↓** | **↓** |  | **↓** |
| **Glycerol** |  | **↑** |  | **↑** | **↓** | **↓** | **↓** | **↓** |
| **Mannitol** | **↓** |  | **↓** |  | **↓** |  | **↓** |  |
| **Myoinositol** | **↓** | **↓** | **↓** | **↓** | **↓** | **↓** | **↓** | **↓** |
| **Propyl alcohol** |  |  |  | **↓** |  |  | **↓** | **↓** |
| **Ribitol** | **↓** | **↓** | **↓** | **↓** | **↓** | **↓** | **↓** | **↓** |
| **Threitol** | **↓** |  | **↑** |  | **↑** |  | **↓** |  |
| **Amino Acids and Derivatives** | | | | | | | | |
| **Acetyllysine** |  | **↓** |  | **↓** |  |  |  | **↓** |
| **Acetyltyrosine** |  | **↓** |  | **↓** | **↓** |  | **↓** | **↓** |
| **Alanine** | **↓** |  | **↓** | **↓** | **↓** | **↓** | **↓** | **↓** |
| **Asparagine** | **↓** |  | **↓** |  | **↓** |  | **↓** |  |
| **Aspartic acid** | **↓** | **↓** | **↓** | **↓** | **↓** | **↓** | **↓** | **↓** |
| **Cystathionine** |  |  |  | **↓** |  | **↓** |  | **↓** |
| **Cysteine** |  |  |  |  |  | **↓** |  | **↓** |
| **Glutamic acid** | **↓** | **↓** | **↓** | **↓** | **↓** | **↓** | **↓** | **↓** |
| **Glutamine** | **↓** | **↓** | **↓** | **↓** | **↓** |  |  |  |
| **Glycine** | **↓** |  | **↓** |  | **↓** |  | **↓** |  |
| **Homocysteine** | **↓** |  | **↓** |  | **↓** | **↓** | **↑** | **↓** |
| **Isoleucine** |  | **↓** | **↑** | **↓** | **↑** | **↓** | **↓** | **↓** |
| **Leucine** | **↓** |  | **↓** |  | **↓** | **↓** | **↑** | **↓** |
| **Lysine** |  | **↓** |  | **↓** |  | **↓** |  | **↓** |
| **Methionine** |  |  |  |  |  | **↓** |  | **↓** |
| **Norvaline** | **↑** | **↓** |  | **↓** | **↑** | **↓** |  | **↓** |
| **Ornithine** |  | **↑** |  | **↑** |  |  |  |  |
| **Phenylalanine** |  | **↓** |  | **↓** |  | **↓** |  | **↓** |
| **Proline** | **↓** | **↓** |  | **↓** | **↓** | **↓** | **↓** | **↓** |
| **Pyroglutamic acid** |  |  |  |  | **↓** | **↑** | **↓** | **↑** |
| **Sarcosine** |  |  |  |  |  |  |  | **↓** |
| **Serine** |  |  |  | **↓** | **↓** |  | **↓** | **↓** |
| **Succinylacetone** | **↓** |  | **↓** |  |  |  |  |  |
| **Threonine** |  | **↓** |  | **↓** |  | **↓** |  | **↓** |
| **Tryptophan** |  | **↓** |  | **↓** |  | **↓** |  | **↓** |
| **Tyrosine** |  | **↓** | **↓** | **↓** | **↓** | **↓** | **↓** | **↓** |
| **Valine** |  |  |  |  |  | **↓** |  | **↓** |
| **Fatty Acids** | | | | | | | | |
| **Linoelaidic acid** |  |  | **↓** |  | **↓** |  | **↓** |  |
| **Oleic acid** | **↓** |  |  |  | **↑** |  | **↓** |  |
| **Palmitic acid** |  |  |  |  |  |  | **↓** |  |
| **Stearic acid** |  |  | **↓** |  | **↓** |  | **↓** |  |
| **Sugars** | | | | | | | | |
| **Acetylglucosamine** | **↑** | **↓** | **↓** | **↓** | **↓** |  | **↓** | **↓** |
| **Arabinose** |  |  |  | **↓** |  | **↓** |  | **↓** |
| **Erythrose** | **↑** |  | **↑** |  | **↑** |  | **↑** |  |
| **Fructose** |  | **↓** |  | **↓** | **↓** | **↓** | **↓** | **↓** |
| **Galactose** | **↓** | **↓** | **↓** | **↓** | **↓** | **↓** | **↑** | **↓** |
| **Glucose** | **↓** | **↓** | **↓** | **↓** | **↓** | **↓** | **↓** | **↓** |
| **Maltose** | **↓** |  | **↓** | **↓** | **↓** | **↓** | **↓** | **↓** |
| **Mannobiose** | **↓** |  |  |  | **↓** |  | **↓** |  |
| **Mannose** |  | **↓** |  | **↓** |  | **↓** |  | **↓** |
| **Methylglucoside** | **↓** | **↓** | **↓** | **↓** | **↓** | **↓** | **↓** | **↓** |
| **Ribose** | **↓** | **↑** | **↓** | **↑** | **↓** | **↓** | **↓** | **↓** |
| **Sedoheptulose** | **↓** | **↓** |  | **↓** | **↓** | **↓** | **↓** | **↓** |
| **Sorbose** |  |  | **↑** |  | **↑** |  | **↑** |  |
| **Tagatose** |  | **↓** |  | **↓** |  | **↓** |  | **↓** |
| **Turanose** | **↓** |  |  |  | **↓** |  | **↓** |  |
| **Xylose** |  | **↓** |  | **↓** |  | **↓** |  | **↓** |
| **Other** | | | | | | | | |
| **Acetaldehyde** |  |  | **↓** |  | **↓** |  | **↓** |  |
| **Adenosine** | **↓** | **↓** |  | **↓** | **↓** | **↓** | **↓** | **↓** |
| **Butanal** |  |  |  |  | **↓** | **↓** |  | **↓** |
| **Cadaverine** |  |  |  | **↓** | **↓** | **↓** | **↓** | **↓** |
| **Cholesterol** |  |  |  |  | **↓** |  |  |  |
| **Decanal** |  |  |  |  | **↑** |  | **↑** |  |
| **Formamide** |  | **↓** |  | **↓** |  | **↓** |  | **↓** |
| **Guanosine** |  |  |  |  |  | **↓** |  |  |
| **Hexane** |  |  |  | **↑** |  | **↑** |  | **↑** |
| **Homolanthionine** |  | **↓** |  | **↓** | **↓** | **↓** | **↓** | **↓** |
| **Hydroxyphenethylamine** |  |  |  | **↓** |  | **↓** |  | **↓** |
| **Indole-3-acetamide** |  | **↓** |  |  |  |  |  |  |
| **Inosine** | **↓** |  | **↓** |  | **↓** | **↓** |  |  |
| **Niacinamide** |  |  |  | **↓** |  | **↓** |  | **↓** |
| **Oleamide** |  |  |  |  | **↓** |  | **↓** |  |
| **Pantothenic acid** |  | **↓** |  | **↓** |  | **↓** |  |  |
| **Pectin** |  |  |  |  | **↓** |  | **↓** |  |
| **Prolylglycine** |  | **↑** |  | **↑** |  |  |  |  |
| **Propanal** | **↓** |  |  | **↓** | **↓** | **↓** |  | **↓** |
| **Propanethiol** |  | **↓** |  | **↓** |  | **↓** |  | **↓** |
| **Purine** |  |  |  |  |  | **↓** |  | **↓** |
| **Putrescine** |  | **↓** |  | **↓** | **↓** | **↓** | **↓** | **↓** |
| **Pyrimidine** |  | **↓** |  | **↓** | **↑** | **↓** | **↓** | **↓** |
| **Pyruvic acid** |  |  |  |  | **↓** |  | **↓** |  |
| **Quinoline** | **↓** |  | **↓** |  | **↓** |  | **↓** |  |
| **Ribonolactone** | **↓** |  |  |  |  | **↓** | **↑** | **↓** |
| **Thiourea** | **↓** |  |  |  | **↓** |  | **↓** |  |
| **Triethylamine** |  | **↓** |  | **↓** |  | **↓** |  | **↓** |
| **Urea** |  | **↑** |  | **↑** |  | **↑** |  | **↑** |
| **Uridine** |  | **↓** |  | **↓** |  |  |  |  |
