# Supplementary material for: Scaled traumatic brain injury results in unique metabolomic signatures between gray matter, white matter, and serum in a piglet model
Source: PLoS One. 2018 Oct 31;13(10):e0206481. doi: 10.1371/journal.pone.0206481 (PMC6209298; doi:10.1371/journal.pone.0206481)
Supplement: S2 Table — Note: ↓ = metabolite decreased after TBI; ↑ = metabolite increased after TBI. (DOCX) [file pone.0206481.s002.docx]

| **Metabolite** | **2m/s; 6mm 24 hours** | **2m/s; 6mm 7 days** | **4m/s; 6mm 24 hours** | **4m/s; 6mm 7 days** | **4m/s; 12mm 24 hours** | **4m/s; 12mm 7 days** | **4m/s; 15mm 24 hours** | **4m/s; 15mm 7 days** |
| --- | --- | --- | --- | --- | --- | --- | --- | --- |
| **Acids** | | | | | | | | |
| **Aminooctanoic acid** |  |  | **↑** |  |  |  | **↑** |  |
| **Citric acid** |  |  |  |  |  | **↓** |  |  |
| **Dihydroxybutanoic acid** |  | **↑** | **↑** | **↑** | **↑** |  | **↑** |  |
| **Homovanillic acid** |  |  |  |  | **↓** |  | **↓** |  |
| **Lactic acid** | **↑** |  | **↑** |  |  |  |  |  |
| **Malic acid** | **↑** |  |  | **↑** |  |  | **↑** | **↑** |
| **Malonic acid** | **↓** | **↓** | **↓** |  | **↓** | **↓** |  | **↓** |
| **p-Hydroxymandelic acid** |  |  | **↓** | **↓** |  |  | **↑** |  |
| **Phosphoric acid** | **↓** |  | **↓** |  | **↓** |  | **↓** |  |
| **Succinic acid** |  |  |  |  | **↓** |  | **↓** | **↓** |
| **Valeric acid** |  | **↑** | **↑** | **↑** | **↑** |  | **↑** | **↑** |
| **Alcohols** | | | | | | | | |
| **Butanediol** | **↓** |  | **↓** |  | **↓** |  | **↓** |  |
| **Galactitol** |  | **↑** | **↑** |  | **↓** | **↑** |  | **↑** |
| **Myoinositol** |  |  |  | **↑** |  |  |  | **↑** |
| **Amino Acids and Derivatives** | | | | | | | | |
| **Alanine** |  | **↓** |  |  |  |  |  | **↓** |
| **Asparagine** |  |  |  |  | **↑** |  | **↑** |  |
| **Aspartic acid** | **↓** | **↑** | **↑** | **↑** |  | **↑** |  | **↑** |
| **Cysteine** |  | **↓** |  |  |  | **↑** |  | **↑** |
| **Glutamine** | **↑** |  |  |  | **↑** |  |  |  |
| **Glycine** | **↓** | **↓** |  | **↓** |  |  | **↓** | **↓** |
| **Isoleucine** | **↑** | **↓** |  | **↓** |  |  | **↑** | **↓** |
| **Leucine** | **↑** | **↓** |  | **↓** | **↑** |  |  | **↓** |
| **Lysine** |  | **↓** |  | **↓** | **↓** |  | **↓** |  |
| **Ornithine** |  | **↑** | **↓** | **↑** | **↓** | **↑** |  |  |
| **Phenylalanine** |  | **↑** |  | **↑** |  |  |  | **↑** |
| **Proline** | **↓** |  | **↓** |  | **↓** |  | **↓** |  |
| **Threonine** | **↑** |  |  |  | **↓** |  |  |  |
| **Tyrosine** |  | **↑** |  | **↑** |  |  |  |  |
| **Valine** |  | **↑** |  |  |  |  |  | **↑** |
| **Creatinine** |  |  | **↑** |  | **↑** |  | **↑** | **↑** |
| **Ketoleucine** |  |  |  |  | **↓** |  | **↓** |  |
| **N-Acetyl-L-aspartic acid** |  |  | **↓** | **↓** | **↓** | **↓** |  | **↓** |
| **Pyroglutamic acid** |  |  |  | **↑** |  |  |  |  |
| **Fatty acids** | | | | | | | | |
| **Hydroxybutyric acid** |  |  | **↑** |  |  |  | **↑** |  |
| **Palmitic acid** | **↓** |  |  |  |  |  | **↓** |  |
| **Sugars** | | | | | | | | |
| **D-arabino-Hexulose 6-phosphate** | **↓** |  | **↑** |  | **↓** |  | **↓** |  |
| **Deoxyribose** | **↑** |  | **↑** |  |  |  |  |  |
| **Fructose** | **↓** | **↓** |  |  | **↓** | **↓** | **↓** | **↓** |
| **Galactose** | **↓** |  |  |  |  |  | **↓** |  |
| **Glucose** | **↓** | **↓** |  | **↓** | **↓** | **↓** |  | **↓** |
| **Ribose** | **↑** |  |  |  | **↓** |  |  |  |
| **Other** | | | | | | | | |
| **Cytosine** |  |  |  |  | **↓** |  | **↓** |  |
| **Guanine** |  |  |  |  | **↓** |  | **↓** |  |
| **Phenylacetamide** | **↓** |  |  |  |  |  | **↓** |  |
| **Phosphate** | **↑** | **↓** |  | **↓** |  |  | **↑** |  |
| **Pyruvic acid** |  | **↓** |  |  |  | **↓** |  | **↓** |
| **Uracil** |  |  | **↓** |  |  |  | **↓** |  |
| **Urea** | **↑** | **↓** | **↑** | **↓** | **↑** | **↓** | **↑** |  |
